# Associations between Dietary Patterns and Impaired Fasting Glucose in Chinese Men: A Cross-Sectional Study

**DOI:** 10.3390/nu7095382

**Published:** 2015-09-21

**Authors:** Meilin Zhang, Yufeng Zhu, Ping Li, Hong Chang, Xuan Wang, Weiqiao Liu, Yuwen Zhang, Guowei Huang

**Affiliations:** 1 Department of Nutrition and Food Science, School of Public Health, Tianjin Medical University, 22 Qixiangtai Road, Heping District, Tianjin 300070, China; E-Mails: defjmmm@163.com (M.Z.); zhuyufeng5580@163.com (Y.Z.); lp900221@gmail.com (P.L.); changhong@tmu.edu.cn (H.C.); wangxuan@tmu.edu.cn (X.W.); 2 Department of Rehabilitation and Sports Medicine, Tianjin Medical University, 22 Qixiangtai Road, Heping District, Tianjin 300070, China; 3 Health Education and Guidance Center of Heping District, 97 Hualong Road, Heping District, Tianjin 300040, China; E-Mails: hptjjs@126.com (W.L.); zyw19830910@gmail.com (Y.Z.)

**Keywords:** dietary pattern, factor analysis, prediabetes, impaired fasting glucose, men, China

## Abstract

Few studies have examined the association between Asian dietary pattern and prediabetes, in particular, the Chinese diet. We conducted a cross-sectional study to identify dietary patterns associated with impaired fasting glucose (IFG) which considered a state of prediabetes in Chinese men. The study included 1495 Chinese men aged 20 to 75 years. Information about diet was obtained using an 81-item food frequency questionnaire (FFQ), and 21 predefined food groups were considered in a factor analysis. Three dietary patterns were generated by factor analysis: (1) a vegetables-fruits pattern; (2) an animal offal-dessert pattern; and (3) a white rice-red meat pattern. The multivariate-adjusted odds ratio (OR) of IFG for the highest tertile of the animal offal-dessert pattern in comparison with the lowest tertile was 3.15 (95% confidence intervals (CI): 1.87–5.30). The vegetables-fruits dietary pattern was negatively associated with the risk of IFG, but a significant association was observed only in the third tertile. There was no significant association between IFG and the white rice-red meat pattern. Our findings indicated that the vegetables-fruits dietary pattern was inversely associated with IFG, whereas the animal offal-dessert pattern was associated with an increased risk of IFG in Chinese men. Further prospective studies are needed to elucidate the diet-prediabetes relationships.

## 1. Introduction

Impaired fasting glucose (IFG) is a common glucose disorder, and considered a state of prediabetes associated with increased risk of diabetes [1] and complications or cardiovascular disease [2,3,4]. Currently, the prevalence of prediabetes in Chinese adults was 15.5%, accounting for 148.2 million adults with prediabetes [5]. More importantly, 5%–10% of people per year with prediabetes will progress to diabetes [6]. Contrary to the relative irreversibility of diabetes, IFG does not typically present with clinical symptoms and can be treated using appropriate dietary intervention measures, thereby delaying or preventing diabetes [7,8]. Currently, many epidemiological studies focus on the dietary pattern and diabetes. Assessing dietary patterns enables the analysis of potentially interactive and antagonistic effects of different nutrients. The healthy balanced dietary pattern, characterized by a diet with a frequent intake of raw and salad vegetables, fruits in both summer and winter, fish, pasta and rice, and low intake of fried foods, sausages, fried fish, and tubers, may be negatively associated with the risk of having undiagnosed diabetes [9]. From the Framingham Offspring Study, it was suggested that consumption of a diet rich in fruits, vegetables, whole grains, and reduced fat dairy protects against insulin resistance phenotypes (impaired glucose tolerance and IFG) and displacing these healthy choices with refined grains, high fat dairy, sweet baked foods, candy, and sugar sweetened soda promotes impaired glucose tolerance and IFG [10]. Few studies have examined the association between Asian dietary pattern and prediabetes, in particular, the Chinese diet. Among Japanese men, a dietary pattern characterized by frequent consumption of dairy products and fruits and vegetables but low alcohol intake may be associated with a decreased risk of developing prediabetes [11]. From the 2002 China National Nutrition and Health Survey, the New Affluence pattern (mainly well-to-do individuals) characterized by living in urban areas, being less physical active, having more smokers, alcohol users, and overweight individuals, and having a higher intake of animal foods and soybean productswas associated with a substantially higher risk of prediabetes in Chinese adults [12].

Due to the rapid economic and social changes, Chinese dietary patterns and lifestyle have changed substantially, we sought to determine the influence of specific dietary patterns on prediabetes in the Chinese population. The objective of this study was to determine the association between various dietary patterns and IFG among Chinese men.

## 2. Materials and Methods

### 2.1. Population

A total of 1615 subjects aged 20–75 years were performed routine health check-up in Health Education and Guidance Center of Heping District, Tianjin, China in 2014. Participants with fasting plasma glucose (FPG) concentration of 110–126 mg/dL (6.1–7.0 mmol/L) were classified as IFG [1], those with a fasting glucose concentration <110 mg/dL (<6.1 mmol/L) were classified as normoglycemic, those with a fasting glucose concentration of ≥126 mg/dL (7.0 mmol/L) or self-reported current diabetes treatments were excluded. The final cross-sectional study population comprised 1459 participants. The study protocol was approved by the Ethics Committee of Tianjin Medical University. Written informed consent was obtained from all participates.

### 2.2. Data Collection

Height was measured without shoes to the nearest 1 cm and weight measured in light clothing to the nearest 0.1 kg on a beam balance scale. Body mass index (BMI) was calculated as weight in kilograms divided by the square of the height in meters (kg/m^2^). Venous blood samples were taken from all participants after an overnight fast (12 h at least) and the samples were stored at −80 °C until assessment assays were performed. Serum total cholesterol (TC) and triglyceride (TG) were measured by routine enzymatic methods. Serum high-density lipoprotein cholesterol (HDL-C) and low-density lipoprotein cholesterol (LDL-C) were measured using colorimetric method. Plasma glucose and lipid levels were determined by automatic biochemical analyzer (TBA-40, Tokyo, Japan).

Information about demographic characteristics and lifestyle habits, including smoking, drinking, and physical activity, were collected by trained interviewers. Current smokers were defined as those who smoked at least one cigarette per day and non-smokers were defined as those who were either former smokers or never smoked a cigarette in their lives. Those who stopped smoking for less than a year were classified as smokers. A participant was classified as “drinker” in case of having drunk beer or any other alcoholic beverage once a week on average during the last year, excluding those who drank beer or any other alcoholic beverage once during festivals.

Physical activity was recorded as a three level variable (light, moderate, and heavy), as recommended by the China Nutrition Society.

### 2.3. Dietary Assessment

Dietary data were collected by trained dietitians during a structured interview. A validated semi-quantitative food frequency questionnaire (FFQ) was used to assess the dietary intake [13]. Participants were asked to report their frequency of consumption of each food item during the past month. The food frequency questionnaire consisted of 81 items, including seven frequency categories as follows: (1) almost never eat or drink; (2) less than once per week; (3) once a week; (4) 2–3 times per week; (5) 4–6 times per week; (6) once a day; (7) twice or more per day. According to the similarity of nutrient profiles and culinary usage among the foods and the grouping scheme used in other studies, we collapsed the 81 food items into 21 predefined food groups. Information on frequency of intake and portion size was used to calculate the amount of each food item consumed on average, using China Food Composition Table (2009) as the database. Four three-day 24-h recalls (24h) in three consecutive days (including two weekdays and one weekend day) were completed to determine the validity of FFQ. Nutrients intake assessed by the FFQ and the average of 24h correlated well, with the correlation coefficients being 0.52–0.64 for macronutrients and 0.29–0.76 for micronutrients.

### 2.4. Statistical Analysis

Descriptive data for study populations are presented as the median (range) for continuous variables and as percentages for categorical variables. To compare the general characteristics according to IFG status, continuous variables were examined using Wilcoxon rank sum test and chi-square test for categorical variables. *P* trend was calculated using generalized linear models for continuous variables. Factor analysis (principal component) was used to extract the participants’ dietary patterns among the 21 predefined food groups. To simplify the interpretation, the factors were rotated by orthogonal transformation (varimax rotation) to maintain uncorrelated factor variables called principal factor or patterns. After evaluating the eigenvalues (≥1.5), screen plot test, and factor interpretability, three factors were retained. Items were retained in a factor if they had an absolute correlation ≥ 0.20 with that factor. Tertiles were categorized across the scores of each dietary pattern based on the distribution of the scores for all the participants and used for further analysis. Relationships between tertile categories of dietary pattern scores and IFG status were examined using logistic regression by three different models. Odds ratios (OR) and corresponding 95% confidence intervals (CI) were calculated. Model 1 was used to calculate the crude OR, and model 2 was adjusted for age, BMI, total energy intake, drinking, smoking, and physical activity. A linear trend across increasing quartiles was tested using the median value of each quartile as a continuous variable based on linear regression. All statistical operations were performed using SPSS Version 19.0 (IBM, Chicago, IL, USA). All reported *P* values were two-sided and a *P* < 0.05 was considered significant.

## 3. Results

The general characteristics of the study population according to IFG status are presented in Table 1. Compared with participants without IFG, participants with IFG tended to be older, have higher BMI, TC, LDL-C, FPG, and TG levels (*p* < 0.05).

**Table 1:** General characteristics of study population.

Characteristics	IFG Status		*p* ^1^
	No (1327)	Yes (132)	
Age (year)	41.0 (32.0–50.0)	50.0 (41.0–58.0)	0.000
BMI (kg/m^2^)	25.1 (23.0–27.1)	26.3 (24.4–28.5)	0.000
TC (mmol/L)	4.8 (4.3–5.3)	5.1 (4.6–5.8)	0.000
TG (mmol/L)	1.4 (0.9–2.0)	1.7 (1.2–2.7)	0.000
HDL-C (mmol/L)	1.1 (1.0–1.3)	1.1 (1.0–1.3)	0.546
LDL-C(mmol/L)	3.0 (2.5–3.5)	3.2 (2.8–3.8)	0.000
FPG(mmol/L)	5.4 (5.2–5.7)	6.4 (6.2–6.6)	0.000
Total energy intake (kcal)	2086.9 (1646.1–2603.7)	2049.1 (1595.1–2481.7)	0.827
Smoking (%)			0.067
Current	41.5	48.5	
Past	12.4	15.9	
Never	46.1	35.6	
Drinking (%)			0.015
Everyday	8.7	16.7	
Sometimes	71.4	66.7	
Past	8.0	9.1	
Never	11.9	7.6	
Physical activity (%)			
Light	80.8	81.0	0.970
Middle	51.5	60.3	0.136
Heavy	35.2	38.8	0.525

IFG, impaired fasting glucose; BMI: Body mass index; TC: total cholesterol; TG: triglycerides; HDL-C: high-density lipoprotein cholesterol; LDL-C: low-density lipoprotein cholesterol; FPG: fasting plasma glucose; ^1^ Analysis of Wilcoxon rank sum test or chi-squared test.

Factor analysis identified three major dietary patterns among the 21 food groups, and the associated factor loading scores with absolute values ≥ 0.20 are shown in Table 2. The “vegetables-fruits” dietary pattern was characterized by high intakes of vegetables, fruits, tubers, seaweeds and mushrooms, coarse cereals, and low intakes of alcohol. The “animal offal-dessert pattern” included high intakes of animal offal, dessert, fast food and beverages. The “white rice-red meat pattern” included high intakes of white rice, red meat, poultry, and eggs. Each dietary pattern explained 16.8%, 8.7%, and 7.8% of the variation in food intake, respectively.

**Table 2:** Factor loadings for the three major dietary patterns derived from principal components analysis with orthogonal rotation.

Foods/Food Groups	Vegetables-Fruits Pattern	Animal Offal-Dessert Pattern	White Rice-Red Meat Pattern
White rice	−0.132	−0.077	0.628
Vegetables	0.698	0.091	0.220
Coarse cereals	0.533	−0.244	0.152
Refined wheat	0.419	−0.116	0.006
Tubers	0.618	0.036	−0.114
Fruits	0.582	0.311	0.086
Seaweeds and mushrooms	0.575	0.250	0.198
Soybean products	0.474	−0.064	0.341
Red meat	−0.050	0.123	0.750
Poultry	0.108	0.161	0.610
Peanuts	0.408	0.230	−0.014
Seafood	0.354	0.454	0.039
Dairy products	0.272	0.115	−0.018
Eggs	0.206	−0.038	0.576
Tea	0.203	0.288	−0.063
Dessert	0.167	0.636	−0.007
Condiments	0.133	0.348	0.236
Animal offal	0.090	0.648	0.115
Alcohol beverages	0.015	0.347	−0.022
Fast food	0.004	0.603	0.021
Beverages	−0.094	0.595	0.053
Variance of explained (%)	16.8	8.7	7.8

Factor loadings with absolute values ≥ 0.20 were listed in the table among 21 food groups.

The distribution of characteristics by dietary pattern score tertiles is presented in Table 3. Increasing scores in the vegetables-fruits patterns were correlated with a decreased percent energy from fat (*p* for trend <0.001), whereas the percent energy from carbohydrate increased as the score of the vegetables-fruits pattern increased. Animal offal-dessert pattern and white rice-red meat pattern, however, has an inverse relationship with the percent energy from fat and carbohydrate. The vegetables-fruits pattern score was associated with a high intake of fiber and low intake of cholesterol, whereas the dessert pattern and white rice-red meat pattern score were associated with a low intake of fiber and high intake of cholesterol. The fatty acids were significantly associated with dietary pattern scores. The vegetables-fruits pattern score was associated with a lower intake of total fatty acids, saturated fatty acid and monounsaturated fatty acids, whereas the animal offal-dessert pattern and white rice-red meat pattern score was positively associated with the intakes of total fatty acids, saturated fatty acid, monounsaturated fatty acids, and polyunsaturated fatty acids. Regarding mineral intake, iron intake was positively associated with the scores of the vegetables-fruits pattern and animal offal-dessert pattern. Magnesium intake was positively associated with the scores of the vegetables-fruits pattern and negatively associated with scores of animal offal-dessert pattern and white rice-red meat pattern. Zinc was positively associated with the scores of the white rice-red meat pattern. Selenium was positively associated with animal offal-dessert pattern.

**Table 3:** Distribution of characteristics by the tertiles of dietary pattern scores.

Characteristics	Vegetables-Fruits Pattern			*p* Trend ^2^
	T1 (*n* = 486) ^1^	T2 (*n* = 486)	T3 (*n* = 487)	
Age (year)	38.5 (32.0–44.0)	41.0 (32.0–51.0)	45.0 (37.0–54.0)	0.000
BMI (kg/m^2^)	25.2 (23.1–27.3)	25.0 (22.9–27.1)	25.2 (23.5–27.2)	0.743
Energy (kcal)	1555.4 (1296.3–1889.5)	2052.6 (1752.7–2363.4)	2687.6 (2321.8–3207.0)	0.000
Fat (g)	50.0 (45.5–54.7)	48.1 (42.4–54.2)	46.8 (38.8–52.7)	0.000
Fat (% energy)	21.0 (18.5–23.9)	19.8 (17.2–22.4)	19.3 (16.3–21.3)	0.000
Protein (g)	89.4 (84.2–94.7)	89.8 (84.8–95.9)	89.0 (82.2–97.8)	0.962
Protein (% energy)	16.9 (15.5–18.3)	16.6 (15.6–17.7)	16.1 (15.1–17.4)	0.000
Carbohydrate (g)	335.0 (312.0–350.5)	340.6 (317.1–358.8)	343.3 (315.6–369.1)	0.000
Carbohydrate (% energy)	59.8 (54.5–64.1)	62.0 (57.4–65.7)	63.1 (59.1–66.8)	0.000
Fiber (g)	19.1 (17.2–20.9)	20.6 (18.3–22.7)	22.9 (20.1–26.5)	0.000
Cholesterol (g)	564.2 (458.3–662.5)	537.5 (446.6–627.1)	498.2 (392.4–610.2)	0.000
Total fatty acids (g)	39.1 (35.1–44.7)	38.8 (32.7–46.2)	38.2 (30.8–46.1)	0.010
SFA (g)	11.7 (10.4–13.9)	11.4 (9.7–13.9)	10.9 (8.8–13.1)	0.000
MUFAs (g)	13.8 (12.3–16.1)	13.4 (11.3–16.0)	12.7 (10.2–15.3)	0.000
PUFAs (g)	10.0 (8.7–11.5)	9.9 (8.0–12.4)	10.1 (7.7–13.6)	0.096
Magnesium, Mg (mg)	444.9 (420.2–465.8)	466.3 (438.1–490.0)	497.3 (461.6–531.0)	0.000
Iron, Fe (mg)	37.8 (35.0–39.8)	37.0 (33.9–39.2)	36.8 (33.6–39.6)	0.001
Mg/Fe ratio	11.6 (11.1–12.4)	12.4 (11.6–13.3)	13.2 (12.4–14.7)	0.000
Zinc, Zn (mg)	14.4 (13.7–15.3)	14.3 (13.5–15.4)	14.4 (13.2–15.6)	0.053
Selenium, Se (mg)	65.4 (58.7–71.0)	63.9 (57.30–70.2)	60.8 (53.6–71.4)	0.088
**Animal giblets-dessert pattern**				
Age (year)	46.0 (38.0–55.0)	41.0 (33.0–50.0)	37.5 (31.0–44.0)	0.000
BMI (kg/m^2^)	25.0 (23.1–27.1)	25.3 (23.1–27.2)	25.1 (23.1–27.2)	0.733
Energy (kcal)	1966.7 (1561.7–2397.1)	1951.0 (1564.9–2434.7)	2324.1 (1913.5–3033.2)	0.000
Fat (g)	44.7 (39.1–50.5)	48.5 (43.9–53.8)	51.4 (46.9–58.3)	0.000
Fat (% energy)	18.2 (15.5–20.9)	20.0 (17.9–22.5)	21.2 (19.3–23.7)	0.000
Protein (g)	89.9 (84.2–95.9)	90.0 (85.2–95.5)	88.5 (82.4–96.4)	0.274
Protein (% energy)	16.7 (15.4–17.9)	16.7 (15.6–17.9)	16.1 (15.0–17.5)	0.000
Carbohydrate (g)	348.0 (324.4–367.2)	336.3 (314.4–353.3)	333.9 (308.5–352.0)	0.000
Carbohydrate (% energy)	63.5 (58.5–67.9)	60.8 (55.9–64.9)	61.2 (56.7–64.5)	0.000
Fiber (g)	21.3 (18.9–23.9)	20.6 (18.4–23.1)	20.0 (17.2–22.5)	0.000
Cholesterol (g)	512.0 (408.8–592.0)	535.9 (444.9–646.8)	550.8 (450.1–668.9)	0.000
Total fatty acids (g)	36.7 (30.9–43.5)	39.3 (34.6–44.7)	40.6 (34.2–49.4)	0.000
SFA (g)	10.8 (9.1–12.8)	11.6 (9.9–13.4)	11.8 (9.8–14.5)	0.000
MUFAs (g)	12.6 (10.5–14.8)	13.6 (11.6–15.5)	14.2 (11.8–16.7)	0.000
PUFAs (g)	9.6 (7.7–12.1)	10.0 (8.5–11.9)	10.5 (8.5–12.7)	0.000
Magnesium, Mg (mg)	481.5 (455.9–508.3)	462.6 (437.1–495.4)	451.0 (413.3–478.6)	0.000
Iron, Fe (mg)	37.3 (34.8–39.5)	37.5 (34.5–39.9)	36.7 (33.1–39.3)	0.002
Mg/Fe ratio	12.7 (11.9–13.7)	12.2 (11.4–13.2)	11.8 (10.9–13.0)	0.000
Zinc, Zn (mg)	14.4 (13.4–15.3)	14.4 (13.6–15.3)	14.4 (13.4–15.7)	0.224
Selenium, Se (mg)	58.7 (53.0–64.9)	63.8 (58.2–69.0)	70.1 (61.9–78.7)	0.000
**White rice-red meat pattern**				
Age (year)	45.0 (36.8–52.0)	41.0 (33.0–51.0)	38.0 (32.0–46.0)	0.000
BMI (kg/m^2^)	25.0 (23.1–27.1)	25.1 (23.2–27.0)	25.3 (23.0–27.5)	0.337
Energy (kcal)	1739.0 (1379.5–2209.7)	1999.7 (1662.4–2485.3)	2440.6 (2052.9–2958.4)	0.000
Fat (g)	44.6 (39.5–49.1)	48.6 (43.3–53.7)	52.4 (47.0–59.9)	0.000
Fat (% energy)	18.1 (15.3–20.6)	20.1 (17.8–22.6)	21.4 (19.4–24.0)	0.000
Protein (g)	84.7 (79.9–88.9)	90.4 (85.1–95.2)	95.3 (88.6–102.9)	0.000
Protein (% energy)	15.6 (14.6–16.7)	16.7 (15.6–17.8)	17.2 (16.1–18.6)	0.000
Carbohydrate (g)	350.7 (335.3–370.6)	337.9 (318.6–358.8)	321.0 (293.8–345.4)	0.000
Carbohydrate (% energy)	64.2 (60.3–68.8)	61.6 (57.6–65.5)	59.1 (54.5–63.4)	0.000
Fiber (g)	21.4 (19.3–23.4)	20.8 (18.5–23.3)	19.3 (16.7–22.5)	0.000
Cholesterol (g)	468.0 (373.0–553.4)	540.6 (453.0–634.3)	603.2 (502.7–752.3)	0.000
Total fatty acids (g)	35.3 (30.3–39.8)	38.9 (33.2–45.1)	43.3 (37.3–51.3)	0.000
SFA (g)	9.8 (8.7–11.2)	11.5(9.9–13.3)	13.5(11.6–15.9)	0.000
MUFAs (g)	11.7 (10.0–13.1)	13.5 (11.6–15.5)	15.6 (13.6–18.3)	0.000
PUFAs (g)	9.6 (7.6–11.3)	10.2 (8.6–12.4)	10.5 (8.5–13.2)	0.000
Magnesium, Mg (mg)	475.9 (451.4–505.0)	466.9 (439.8–495.6)	450.9 (415.0–482.3)	0.000
Iron, Fe (mg)	37.2 (34.5–39.4)	37.4 (34.4–39.7)	37.0 (34.2–39.5)	0.871
Mg/Fe ratio	12.7 (11.8–13.7)	12.4 (11.4–13.4)	11.9 (11.1–13.0)	0.000
Zinc, Zn (mg)	13.5 (12.7–14.1)	14.4 (13.7–15.1)	15.6 (14.7–16.6)	0.000
Selenium, Se (mg)	64.3 (57.4–70.9)	64.5 (57.2–70.8)	62.0 (54.9–70.5)	0.835

^1^ Tertiles of dietary pattern scores; ^2^ *p* trend was calculated using generalized linear models for continuous variables; *p* trend of nutrient consumption was adjusted for total energy intake. Abbreviation: SFA: saturated fatty acids; PUFAs: polyunsaturated fatty acids; MUFAs: monounsaturated fatty acids; BMI: Body mass index.

The ORs and 95% CIs of IFG were analyzed across the tertiles of dietary pattern scores (Table 4). The OR (95% CI) in the highest tertiles of the vegetables-fruits dietary pattern compared to those in the lowest tertiles in crude model was 1.15 (0.75–1.75), but a significant association was observed in multivariate model 2 (OR: 0.57, 95% CI: 0.34–0.95). Population in the highest tertile of the animal offal-dessert pattern score had an increased risk of IFG in the multivariate-adjusted models when compared with those in the lowest tertile (OR: 2.89, 95% CI: 1.76–4.75) in the multivariate model and this association was stronger (OR: 3.15, 95% CI: 1.87–5.30) in multivariate model 2. The white rice-red meat pattern was not significantly associated with IFG in any of the models.

**Table 4:** Distribution of characteristics by the tertiles of dietary pattern scores.

Dietary Pattern		No. of IFG	Crude Model	Multivatiate Model 1 ^1^	Multivatiate Model 2 ^2^
Vegetables-fruits pattern	T1 ^3^	45	1.00	1.00	1.00
	T2	36	0.78 (0.50–1.24)	0.63 (0.39–1.02)	0.57 (0.31–1.09)
	T3	51	1.15 (0.75–1.75)	0.73 (0.46–1.15)	0.57 (0.34–0.95)
	*p* trend ^4^		0.151	0.700	0.013
Animal offal-dessert pattern	T1	37	1.00	1.00	1.00
	T2	45	1.24 (0.78–1.95)	1.84 (1.13–2.99)	1.86 (1.14–3.02)
	T3	50	1.39 (0.89–2.17)	2.89 (1.76–4.75)	3.15 (1.87–5.30)
	*p* trend		0.370	0.001	0.035
White rice-red meat pattern	T1	49	1.00	1.00	1.00
	T2	48	0.98 (0.64–1.49)	1.14 (0.74–1.76)	1.13 (0.73–1.76)
	T3	35	0.69 (0.44–1.10)	0.91 (0.59–1.53)	0.92 (0.55–1.52)
	*p* trend		0.095	0.942	0.557

^1^ Adjusted for age and body mass index; ^2^ Model 1 + additional adjustment for total energy intake, drinking status, smoking status, physical activity status; ^3^ Tertiles of dietary pattern scores; ^4^ Tests for trend were conducted by assigning the median value to each tertile of food intake as a continuous variable. Abbreviation: IFG, impaired fasting glucose.

## 4. Discussion

We examined associations between dietary patterns and IFG. We identified three major dietary patterns: a vegetables-fruits food pattern (rich in vegetables, fruits, tubers, seaweeds and mushrooms, coarse cereals, and low intake of alcohol), an animal offal-dessert pattern (rich in animal offal, dessert, fast food, and beverages) and a white rice-red meat pattern(rich in white rice, red meat, poultry, and eggs). A vegetables-fruits food pattern and an animal offal-dessert food pattern were associated with IFG; however, a white rice-red meat food pattern was not associated with IFG.

To the best of our knowledge, there have been few studies published that have examined the relationship between dietary patterns, derived by factor analysis, and IFG [9,10,11,12]. The prudent dietary pattern, which was rich in fruits and vegetables [9,14], particularly the high green leafy vegetables intake [15], was associated with a reduced risk of type 2 diabetes. In the Da Qing study for diabetes prevention conducted in China, a diet high in vegetables and fruits was associated with a lower incidence of diabetes [16]. The preventive effects of fruits and vegetables have been hypothesized to be mediated by antioxidants [17], and results of some follow-up studies have supported this hypothesis [18,19,20]. Our findings on the relation between the dietary pattern reflecting vegetable and fruit intake and prediabetes risk is in line with results of previous studies, which have shown an inverse association between consumption of fruits and vegetables and the risk of type 2 diabetes. Our results also showed that the vegetables-fruits pattern score was associated with high intakes of magnesium and iron. Magnesium is an essential cofactor for multiple enzymes involved in glucose metabolism and has been discovered to play a role in the development of diabetes [21]. Animal studies have shown that low magnesium diet can lead to impaired insulin secretion and action [22] and magnesium supplementation decrease the incidence of diabetes [23]. The green leafy vegetables have a high content of magnesium [24], which could reduce the risk of prediabetes and diabetes. In the Chinese population, the main source of iron is from plant foods, which contain mainly non-heme iron. Iron status is associated with diabetes in China [25]. Iron overload may stimulate oxidative stress and inflammation, thus promoting the development of diabetes [26,27]. The intake of iron was positively related to the risk of diabetes, whilst magnesium intake was inversely related. Although our results showed that the vegetables-fruits pattern score was associated with high intakes of iron, a strong inverse association between magnesium: iron intake ratio and diabetes could explain this pattern was negatively associated with IFG [28]. In our study, it was shown that the magnesium: iron intake ratio was increased across the tertile in this pattern. Moreover, vegetables and fruits are rich in dietary fiber, which is a known protective factor for diabetes. Recently, Frank *et al.* found a dietary pattern among urban Ghanaian population, which was characterized by a high consumption of plantain, cassava, and garden egg, and a low intake of rice, juice, vegetable oil, eggs, chocolate drink, sweets, and red meat was related to higher serum triglyceride concentrations and increased the risk of type 2 diabetes [29]. However, an inverse association between cassava flour and incident diabetes was also observed in a Brazilian study [30]. As for cassava, a major carbohydrate source in Africa, contains potentially diabetogenic chemicals, however, its consumption could be considered in diets for the prevention and control of diabetes. The preparation methods were diverse including cooking, frying, and pounding. The different associations of cassava with diabetes could be explained by novel methods for the preparation of the cassava. Alternatively, the combined consumption of cassava and rice with other food groups [31] may represent dietary diversity, which is inversely associated with biomarkers of type 2 diabetes [32]. In the present study, however, the tubers in this dietary pattern were not apparently associated with IFG. The lack of an association may reflect low consumption of tubers in the Chinese men; mean daily intake is 52.9 g for tubers (Appendix).

This study also found that an animal offal-dessert pattern characterized by high intake of animal offal, dessert, fast food and beverages was positively associated with IFG. The animal offal are generally high in saturated fatty acid, cholesterol, iron, and selenium, and desserts contribute importantly to glycemic load (GL), which appear to be associated with increased IFG risk. In the Nurses’ Health Study (NHS), Schulze et al. observed a pattern, which was high in sugar-sweetened soft drinks, refined grains, diet soft drinks, and processed meat but low in wine, coffee, cruciferous vegetables, and yellow vegetables, was associated with an increased risk of diabetes [33]. Desserts and sweets have been found to be part of the Western dietary pattern associated with higher diabetes risk [34,35]. In addition, our study showed that the animal offal-dessert pattern score was associated with a low intake of magnesium and a higher intake of selenium and iron. A cross-sectional study in Chinese population explored a significant positive correlation between dietary selenium intake and the prevalence of diabetes [36]. The findings of our study suggest a significant positive association between dietary selenium intake and animal offal-dessert pattern, consistent with the conclusions of studies on associations between dietary selenium and diabetes [37], supplementary selenium and diabetes [38], and serum selenium and diabetes [39,40]. An increased level of dietary selenium intake may increase the release of glucagon, which can lead to hyperglycemia [41]. Moreover, a high level of dietary selenium intake may stimulate the release of glucagon or may induce overexpression of glutathione peroxidase 1 (GPx1), which is a type of antioxidant selenoprotein. The high activity of GPx1 can interfere with insulin signaling, which is critical to the regulation of glucose levels and the prevention of diabetes [42]. Thus, the animal offal-dessert pattern was associated with an increased risk of IFG in our study.

Our findings demonstrated that the white rice-red meat pattern characterized by white rice, red meat, poultry, and eggs was not associated with IFG. In China, white rice is a staple food and major source of calories contributing to a high dietary GL. The cooked rice, congee (rice porridge) and rice noodle are the three most common white rice-based foods. The Shanghai Women’s Health Study has linked higher dietary GL, primarily from white rice, to an increased risk of diabetes [43]. However, the association between rice intake and diabetes has been inconsistent in China [44,45]. Study in a single province (Jiangsu China) observed a significantly positive association between white rice intake and hyperglycemia risk, whereas no association was observed in a Hong Kong population [45]. Compared with the refined grains (such as white rice), the coarse cereals were low in glycemic index (GI) and rich in fiber may lower the risk of diabetes [46]. It was suggested that high intakes of refined grains intake and usual diets with high GL diet were associated with reduced β-cell function in pre-diabetic Japanese-Brazilians [47]. The updated analyses from three large US cohorts and meta analyses provide evidence that higher dietary GI and GL are associated with increased risk of diabetes, participants who consumed diets with high GI or high GL and low cereal fiber had a nearly 40% higher risk of compared with those whose diets were high in cereal fiber and low in GI or GL [48]. A systematic review and meta-analysis of cohort studies of meat consumption and diabetes risks suggested that meat consumption increased the risk of diabetes [49], particularly processed red meat was associated with an increased risk of diabetes [50]. Eggs, generally regarded as an important dietary source of protein, are commonly consumed in China. Findings from some studies suggest that dietary cholesterol from eggs leads to a modest increase in blood concentrations of total and LDL-C [51,52,53], which have been found to be positively related to diabetes risk [52,54]. On the other hand, eggs contain many other potentially beneficial nutrients, such as monounsaturated fats, minerals, essential amino acids, folate, and other B vitamins. Moreover, consumption of eggs instead of carbohydrate-rich foods may raise HDL-C levels and decrease blood glycemic and insulinemic responses [55]. The data from a representative sample in Jiangsu Province, China indicate that consumption of more than 1 egg/day is associated with significantly elevated risk for diabetes [56]. It was reported that the traditional south pattern of rice as the major staple food in conjunction with pork and vegetable dishes was associated with lower risk of general and abdominal obesity [57]. The rice rich “traditional” pattern was associated with reduced weight gain in Jiangsu province of China [58]. Moreover, the “Green Water” dietary pattern, characterized by high intakes of rice and vegetables and moderate intakes in animal foods was related to the lowest prevalence of metabolic syndrome (MS) (15.9%) in a large-scale, nationally-representative sample of Chinese adults [59]. However, our study showed that there was no relationship between white rice-red meat pattern and IFG. On one hand, the factor loading of white rice in this pattern derived from principal components analysis was 0.628, which was higher than the factor loading of coarse cereals (0.152). Although the white rice contains higher dietary GI and GL, the consumption of white rice in our study was 131.7 g/day (Appendix), which was lower than the southern city. The prospective study conducted in Shanghai, China, reported a diabetes relative risk of 1.78 (95% CI: 1.48–2.15) comparing middle-aged women who consumed ≥300 g/day (versus <200 g/day) of white rice [43]. On the other hand, although the higher contribution to this dietary pattern score was red meat, poultry, and egg, the lack of an association may reflect moderate consumption of red meat in the Chinese men; the mean daily intake was 49.3 g for red meat, 25.1 g for poultry and 62.5 g for egg (Appendix). Additionally, we found the white rice-red meat pattern score was associated with a low intake of magnesium and high intake of zinc. Zinc content is highest in animal-source foods, relatively high in whole-grain cereals, and low in refined cereals and vegetables [60]. Zinc has been postulated that zinc deficiency may aggravate the insulin resistance in noninsulin dependent diabetes mellitus over a six year follow-up study [61]. From above we did not conclude the white rice-red meat pattern score was associated with IFG. Further study will be performed to determine the casual role of white rice-red meat pattern and IFG.

Several potential limitations of this study need to be highlighted. First, because of the cross-sectional design of our study, we cannot assess the causal relationships between dietary patterns and the risk of IFG. Second, because of the nature of the self-reporting questionnaire, recall bias exists and the food intake may be not exact. Third, oral glucose tolerance tests were not performed, possibly leading to an underestimate of the diagnosis of prediabetes. Finally, even though we have adjusted for confounders that related prediabetes risk, we cannot rule out that unmeasured factors have influenced our observed results. The health checkup center-based design leads to a study population that is not fully representative of the general one, and thus, the generalizability of our findings may be questionable. In addition, the lack of racial diversity limits our ability to generalize our results to other populations. Despite these limitations, this study could reveal the relationships between dietary patterns and IFG in Chinese men.

## 5. Conclusions

The vegetables-fruits dietary pattern, which was characterized by high intakes of fruits and vegetables, and low consumption of alcohol, appears to be negatively associated with IFG in Chinese men. Whereas, the animal offal-dessert pattern, which was characterized by frequent consumption of animal offal and dessert might be associated with an increased risk of developing IFG in Chinese men. Further large prospective epidemiologic studies to investigate the effect of those dietary patterns on IFG in Chinese populations are needed in the future.

## Appendix

**Table A1:** Intakes of selected dietary items according to tertiles of dietary pattern score among Chinese men.

Dietary Items	All subjects	Vegetables-Fruits Pattern			Animal offal-Dessert Pattern			White Rice-Red Meat Pattern		
		T1 ^1^	T2	T3	T1	T2	T3	T1	T2	T3
White rice (g)	131.7 (128.7–184.4)	131.7 (128.7–360.4)	141.8 (128.7–184.4)	131.7 (65.9–184.4)	180.2 (128.7–198.5)	131.7 (128.7–184.4)	131.7 (65.9–184.4)	70.9 (64.4–131.7)	131.7 (128.7–184.4)	198.5 (180.2–368.8)
Vegetables (g)	309.7 (217.6–436.5)	209.4 (155.6–282.4)	304.5 (236.4–382.6)	458.0 (361.3–612.6)	308.6 (216.5–441.7)	291.9 (210.8–410.8)	337.8 (230.6–457.5)	278.9 (195.8–384.3)	304.2 (215.4–420.3)	358.7 (262.2–488.9)
Coarse cereals (g)	82.4 (47.3–130.6)	46.7 (18.7–82.4)	82.4 (54.7–129.0)	129.0 (82.4–188.7)	116.7 (60.9–166.4)	76.8 (46.7–129.0)	74.6 (32.9–116.7)	76.8 (32.9–116.7)	82.4 (53.8–129.0)	107.6 (53.8–164.7)
Refined wheat (g)	140.8 (94.5–196.4)	95.4 (67.9–135.8)	147.8 (107.0–194.4)	185.0 (135.5–272.6)	147.0 (96.3–194.6)	137.5 (93.0–188.2)	140.2 (94.5–206.3)	147.0 (95.4–204.8)	143.1 (95.5–191.2)	136.3 (92.2–194.4)
Tubers (g)	52.9 (28.3–94.0)	28.3 (17.9–45.1)	52.9 (35.6–64.9)	100.5 (52.9–141.6)	52.0 (28.2–93.8)	45.1 (27.3–82.8)	52.9 (28.3–100.5)	45.1 (27.8–100.5)	52.9 (28.3–82.8)	51.1 (28.3–82.8)
Fruits (g)	228.2 (134.5–344.2)	136.5 (89.8–210.7)	230.6 (164.5–311.1)	341.1 (241.3–473.2)	198.3 (110.8–296.0)	203.7 (120.3–308.8)	281.9 (182.2–418.2)	201.0 (121.6–336.5)	236.1 (126.7–339.2)	242.9 (147.5–355.2)
Seaweeds and mushrooms (g)	15.7 (8.6–27.3)	8.6 (6.1–13.2)	15.7 (8.6–25.0)	28.6 (15.7–39.3)	12.1 (7.9–25.0)	13.2 (7.9–25.0)	21.4 (12.1–30.3)	12.1 (7.9–25.0)	13.2 (8.6–28.6)	17.5 (10.3–28.6)
Soybean products (g)	78.8 (46.1–127.7)	46.4 (27.1–77.6)	78.8 (52.4–120.3)	103.1 (78.8–152.2)	81.2 (46.4–139.3)	78.8 (46.4–126.0)	77.5 (45.1–104.0)	64.5 (36.1–82.3)	78.8 (46.4–116.5)	93.7 (68.8–150.7)
Red meat (g)	49.3 (31.9–81.6)	41.0 (33.4–74.8)	41.0 (33.4–85.3)	51.7 (31.9–81.6)	37.4 (19.9–71.3)	41.0 (33.8–74.8)	67.4 (37.4–85.6)	31.9 (13.9–37.4)	51.7 (34.0–71.3)	85.6 (67.7–107.2)
Poultry (g)	25.1 (10.1–50.3)	25.1 (10.1–30.2)	25.1 (10.1–30.2)	25.1 (12.1–50.3)	25.1 (10.1–30.2)	25.1 (10.1–30.2)	25.1 (12.1–50.3)	12.1 (6.0–25.1)	25.1 (11.4–30.2)	50.3 (25.1–70.4)
Peanuts (g)	11.1 (6.9–23.6)	6.9 (0.0–11.1)	11.1 (6.9–23.6)	23.6 (8.3–38.7)	6.9 (0.0–20.9)	8.3 (6.9–23.6)	13.9 (6.9–34.7)	11.1 (6.6–23.6)	11.1 (6.9–26.4)	8.3 (4.2–23.6)
Seafood (g)	25.7 (17.6–43.0)	22.2 (11.8–32.5)	25.1 (19.7–43.0)	38.2 (22.2–69.3)	21.5 (10.9–34.2)	23.7 (18.6–40.8)	38.9 (22.2–69.3)	25.7 (17.1–43.0)	24.2 (18.6–44.3)	27.0 (18.6–44.3)
Dairy products (g)	78.3 (17.9–178.6)	48.7 (10.5–95.5)	90.7 (21.0–178.6)	114.7 (46.2–199.6)	46.2 (17.9–146.9)	70.3 (19.1–148.0)	95.5 (48.7–191.1)	77.2 (17.9–178.6)	86.6 (19.1–178.6)	78.3 (17.9–147.0)
Eggs (g)	62.5 (36.6–77.1)	56.3 (28.4–73.7)	66.4 (49.3–77.1)	69.0 (50.3–78.0)	69.0 (49.3–73.7)	66.4 (49.3–77.1)	57.2 (31.2–78.0)	47.4 (25.9–61.2)	61.5 (49.3–74.3)	77.1 (69.0–94.2)
Tea (mL)	192.9 (21.4–364.3)	107.1 (0.0–300.0)	171.4 (37.5–342.9)	300.0 (42.9–600.0)	96.4 (0.0–321.4)	171.4 (42.9–342.9)	257.1 (85.7–600.0)	182.1 (42.9–428.6)	214.3 (42.9–385.7)	150.0 (0.0–342.9)
Dessert (g)	14.4 (3.1–29.4)	13.2 (3.1–23.2)	13.9 (3.1–25.4)	17.5 (3.1–43.2)	3.1 (0.0–11.0)	14.4 (4.3–22.8)	32.3 (17.5–57.0)	13.2 (0.0–27.7)	15.2 (3.1–26.5)	15.3 (3.1–30.6)
Condiments (g)	14.3 (3.5–29.6)	12.9 (2.5–29.4)	14.5 (2.9–29.3)	16.4 (5.1–31.9)	6.8 (1.4–17.8)	14.3 (3.9–29.3)	25.1 (13.2–32.5)	8.4 (2.5–17.8)	15.7 (4.2–29.6)	20.0 (5.1–32.5)
Animal offal (g)	6.4 (0.0–15.3)	6.4 (0.0–15.3)	6.4 (0.0–15.3)	6.4 (0.0–21.6)	0.0 (0.0–6.4)	6.4 (0.0–13.5)	20.6 (7.9–40.7)	6.4 (0.0–15.3)	6.4 (0.0–15.3)	8.1 (0.0–19.9)
Alcohol beverages (mL)	71.4 (0.0–233.9)	65.9 (0.0–205.4)	61.8 (0.0–222.6)	83.2 (0.0–273.6)	29.6 (0.0–107.7)	79.6 (14.3–250.0)	148.2 (29.6–367.9)	79.1 (0.0–250.0)	65.4 (0.0–219.7)	65.4 (0.0–219.6)
Fast food (g)	18.6 (5.3–42.6)	17.5 (5.3–37.3)	18.6 (5.3–38.4)	19.4 (5.3–47.9)	5.3 (0.0–14.9)	18.6 (8.9–32.3)	45.7 (24.6–74.6)	17.0 (0.0–37.6)	19.3 (5.3–42.2)	19.3 (5.3–45.7)
Beverages (mL)	27.7 (0.0–84.0)	41.1 (0.0–84.0)	26.8 (0.0–83.1)	26.8 (0.0–82.1)	0.0 (0.0–26.8)	27.7 (0.0–68.8)	84.0 (29.5–206.3)	26.8 (0.0–82.1)	26.8 (0.0–81.2)	31.4 (0.0–133.9)

^1^ Tertiles of dietary pattern scores.

## References

[1] American Diabetes Association (2012). Standards of medical care in diabetes-2012. Diabetes Care.

[2] Schmidt M.I., Duncan B.B., Bang H., Pankow J.S., Ballantyne C.M., Golden S.H., Folsom A.R., Chambless L.E. (2005). Identifying individuals at high risk for diabetes—The Atherosclerosis Risk in Communities study. Diabetes Care.

[3] Levitzky Y.S., Pencina M.J., D’Agostino R.B., Meigs J.B., Murabito J.M., Vasan R.S., Fox C.S. (2008). Impact of impaired fasting glucose on cardiovascular disease. J. Am. Coll. Cardiol..

[4] Ford E.S., Zhao G.X., Li C.Y. (2010). Pre-Diabetes and the risk for cardiovascular disease asystematic review of the evidence. J. Am. Coll. Cardiol..

[5] Xu Y., Wang L.M., He J., Bi Y.F., Li M., Wang T.G., Wang L.H., Jiang Y., Dai M., Lu J.L. (2013). Prevalence and Control of Diabetes in Chinese Adults. J. Am. Med. Assoc..

[6] Tabak A.G., Herder C., Rathmann W., Brunner E.J., Kivimaki M. (2012). Prediabetes: A high-risk state for diabetes development. Lancet.

[7] Diabetes Prevention Program Research Group (2002). Reduction in the incidence of type 2 diabetes with lifestyle intervention or metformin. N. Engl. J. Med..

[8] Perreault L., Pan Q., Mather K.J., Watson K.E., Hamman R.F., Kahn S.E., Diabetes Prevention Program Research Group (2012). Effect of regression from prediabetes to normal glucose regulation on long-term reduction in diabetes risk: Results from the Diabetes Prevention Program Outcomes Study. Lancet.

[9] Williams D.E., Prevost A.T., Whichelow M.J., Cox B.D., Day N.E., Wareham N.J. (2000). A cross-sectional study of dietary patterns with glucose intolerance and other features of the metabolic syndrome. Br. J. Nutr..

[10] Liu E., McKeown N.M., Newby P.K., Meigs J.B., Vasan R.S., Quatromoni P.A., D’ Agostino R.B., Jacques P.F. (2009). Cross-sectional association of dietary patterns with insulin-resistant phenotype among adults without diabetes in the Framinham Offspring study. Br. J. Nutr..

[11] Mizoue T., Yamaji T., Tabata S., Yamaguchi K., Ogawa S., Mineshita M., Kono S. (2006). Dietary patterns and glucose tolerance abnormalities in Japanese men. J. Nutr..

[12] He Y.N., Hu Y., Ma G., Feskens E.J., Zhai F., Yang X., Li Y. (2009). Dietary Patterns and Glucose Tolerance Abnormalities in Chinese Adults. Diabetes Care.

[13] Jia Q., Xia Y., Zhang Q., Wu H., Du H., Liu L., Wang C., Shi H., Guo X., Liu X. (2015). Dietary patterns are associated with prevalence of fatty liver disease in adults. Eur. J. Clin. Nutr..

[14] Montonen J., Knekt P., Harkanen T., Jarvinen R., Heliovaara M., Aromaa A., Reunanen A. (2005). Dietary patterns and the incidence of type 2 diabetes. Am. J. Epidemiol..

[15] Li M., Fan Y., Zhang X., Hou W., Tang Z. (2014). Fruit and vegetable intake and risk of type 2 diabetes mellitus: Meta-analysis of prospective cohort studies. BMJ Open.

[16] Pan X.R., Li G.W., Hu Y.H., Wang J.X., Yang W.Y., An Z.X., Hu Z.X., Lin J., Xiao J.Z., Cao H.B. (1997). Effects of diet and exercise in preventing NIDDM in people with impaired glucose tolerance: The Da Qing IGT and diabetes study. Diabetes Care.

[17] Hamer M., Chida Y. (2007). Intake of fruit, vegetables, and antioxidants and risk of type 2 diabetes: Systematic review and meta-analysis. J. Hypertens..

[18] Feskens E.J., Virtanen S.M., Räsänen L., Tuomilehto J., Stengård J., Pekkanen J., Nissinen A., Kromhout D. (1995). Dietary factors determining diabetes and impaired glucose tolerance: A 20-year follow-up of the Finnish and Dutch cohorts of the Seven Countries Study. Diabetes Care.

[19] Salonen J.T., Nyyssönen K., Tuomainen T.P., Mäenpää P.H., Korpela H., Kaplan G.A., Lynch J., Helmrich S.P., Salonen R. (1995). Increased risk of non-insulin dependent diabetes mellitus at low plasma vitamin E concentrations: A four year follow up study in men. BMJ.

[20] Reunanen A., Knekt P., Aaran R.K., Aromaa A. (1998). Serum antioxidants and risk of non-insulin dependent diabetes mellitus. Eur. J. Clin. Nutr..

[21] Barbagallo M., Dominguez L.J., Galioto A., Ferlisi A., Cani C., Malfa L., Pineo A., Busardo A., Paolisso G. (2003). Role of magnesium in insulin action, diabetes and cardio-metabolic syndrome X. Mol. Aspects. Med..

[22] Suarez A., Pulido N., Casla A., Casanova B., Arrieta F.J., Rovira A. (1995). Impaired tyrosine-kinase activity of muscle insulin receptors from hypomagnesaemic rats. Diabetologia.

[23] Balon T.W., Gu J.L., Tokuyama Y., Jasman A.P., Nadler J.L. (1995). Magnesium supplementation reduces development of diabetes in a rat model of spontaneous NIDDM. Am. J. Physiol..

[24] Lopez-Ridaura R., Willett W.C., Rimm E.B., Liu S., Stampfer M.J., Manson J.E., Hu F.B. (2004). Magnesium intake and risk of type 2 diabetes in men and women. Diabetes Care.

[25] Shi Z., Hu X., Yuan B., Pan X., Meyer H.E., Holmboe-Ottesen G. (2006). Association between serum ferritin, hemoglobin, iron intake, and diabetes in adults in Jiangsu, China. Diabetes Care.

[26] Fernandez-Real J.M., Lopez-Bermejo A., Ricart W. (2002). Cross-talk between iron metabolism and diabetes. Diabetes.

[27] Swaminathan S., Fonseca V.A., Alam M.G., Shah S.V. (2007). The role of iron in diabetes and its complications. Diabetes Care.

[28] Shi Z.M., Hu X.S., Yuan B.J., Gibson R., Dai Y., Garg M. (2008). Association between magnesium: Iron intake ratio and diabetes in Chinese adults in Jiangsu Province. Diabet. Med..

[29] Frank L.K., Jannasch F., Kröger J., Bedu-Addo G., Mockenhaupt F.P., Schulze M.B., Danquah I. (2015). A dietary pattern derived by reduced rank regression is associated with type 2 diabetes in an urban Ghanaian population. Nutrients.

[30] Rosa M.L., Falcão P.M., Yokoo E.M., da Cruz Filho R.A., Alcoforado V.M., de Souza Bda S., Pinto F.N., Nery A.B. (2014). Brazil’s staple food and incidents diabetes. Nutrition.

[31] Frank L.K., Kroger J., Schulze M.B., Bedu-Addo G., Mockenhaupt F.P., Danquah I. (2014). Dietary patterns in urban Ghana and risk of type 2 diabetes. Br. J. Nutr..

[32] Kant A.K., Graubard B.I. (2005). A comparison of three dietary pattern indexes for predicting biomarkers of diet and disease. J. Am. Coll. Nutr..

[33] Schulze M.B., Hoffmann K., Manson J.E., Willett W.C., Meigs J.B., Weikert C., Heidemann C., Colditz G.A., Hu F.B. (2005). Dietary pattern, inflammation, and incidence of type 2 diabetes in women. Am. J. Clin. Nutr..

[34] Fung T.T., Schulze M., Manson J.E., Willett W.C., Hu F.B. (2004). Dietary patterns, meat intake, and the risk of type 2 diabetes in women. Arch. Intern. Med..

[35] Van Dam R.M., Rimm E.B., Willett W.C., Stampfer M.J., Hu F.B. (2002). Dietary patterns and risk for type 2 diabetes mellitus in U.S. men. Ann. Intern. Med..

[36] Wei J., Zeng C., Gong Q.Y., Yang H.B., Li X.X., Lei G.H., Yang T.B. (2015). The association between dietary selenium intake and diabetes: A cross-sectional study among middle-aged and older adults. Nutr. J..

[37] Stranges S., Sieri S., Vinceti M., Grioni S., Guallar E., Laclaustra M., Muti P., Berrino F., Krogh V. (2010). A prospective study of dietary selenium intake and risk of type 2 diabetes. BMC Public Health..

[38] Stranges S., Marshall J.R., Natarajan R., Donahue R.P., Trevisan M., Combs G.F., Cappuccio F.P., Ceriello A., Reid M.E. (2007). Effects of long-term selenium supplementation on the incidence of type 2 diabetes: A randomized trial. Ann. Intern. Med..

[39] Bleys J., Navas-Acien A., Guallar E. (2007). Serum selenium and diabetes in U.S. adults. Diabetes Care.

[40] Laclaustra M., Navas-Acien A., Stranges S., Ordovas J.M., Guallar E. (2009). Serum selenium concentrations and diabetes in U.S. adults: National Health and Nutrition Examination Survey (NHANES) 2003–2004. Environ. Health. Perspect..

[41] Satyanarayana S., Sekhar J.R., Kumar K.E., Shannika L.B., Rajanna B., Rajanna S. (2006). Influence of selenium (antioxidant) on gliclazide induced hypoglycaemia/anti hyperglycaemia in normal/alloxan-induced diabetic rats. Mol. Cell. Biochem..

[42] Goldstein B.J., Mahadev K., Wu X. (2005). Redox paradox: Insulin action is facilitated by insulin-stimulated reactive oxygen species with multiple potential signaling targets. Diabetes.

[43] Villegas R., Liu S., Gao Y.T., Yang G., Li H., Zheng W., Shu X.O. (2007). Prospective study of dietary carbohydrates, glycemic index, glycemic load, and incidence of type 2 diabetes mellitus in middle-aged Chinese women. Arch. Intern. Med..

[44] Shi Z., Taylor A.W., Hu G., Gill T., Wittert G.A. (2012). Rice intake, weight change and risk of the metabolic syndrome development among Chinese adults: The Jiangsu Nutrition Study (JIN). Asia Pac. J. Clin. Nutr..

[45] Yu R., Woo J., Chan R., Sham A., Ho S., Tso A., Cheung B., Lam T.H., Lam K. (2011). Relationship between dietary intake and the development of type 2 diabetes in a Chinese population: The Hong Kong Dietary Survey. Public Health Nutr..

[46] Kaur K.D., Jha A., Sabikhi L., Singh A.K. (2014). Significance of coarse cereals in health and nutrition: A review. J. Food Sci. Technol..

[47] Sartorelli D.S., Franco L.J., Damião R., Gimeno S., Cardoso M.A., Ferreira S.R. (2009). Dietary glycemic load, glycemic index, and refined grains intake are associated with reduced beta-cell function in prediabetic Japanese migrants. Arq. Bras. Endocrinol. Metabol..

[48] Bhupathiraju S.N., Tobias D.K., Malik V.S., Pan A., Hruby A., Manson J.E., Willett W.C., Hu F.B. (2014). Glycemic index, glycemic load, and risk of type 2 diabetes: Results from 3 large US cohorts and an updated meta-analysis. Am. J. Clin. Nutr..

[49] Aune D., Ursin G., Veierod M.B. (2009). Meat consumption and the risk of type 2 diabetes: A systematic review and meta-analysis of cohort studies. Diabetologia.

[50] Pan A., Sun Q., Bernstein A.M., Schulze M.B., Manson J.E., Willett W.C., Hu F.B. (2011). Red meat consumption and risk of type 2 diabetes: 3 cohorts of US adults and an updated meta-analysis. Am. J. Clin. Nutr..

[51] Nakamura Y., Iso H., Kita Y., Ueshima H., Okada K., Konishi M., Inoue M., Tsugane S. (2006). Egg consumption, serum total cholesterol concentrations and coronary heart disease incidence: Japan Public Health Center-based prospective study. Br. J. Nutr..

[52] Howell W.H., McNamara D.J., Tosca M.A., Smith B.T., Gaines J.A. (1997). Plasma lipid and lipoprotein responses to dietary fat and cholesterol: A meta-analysis. Am. J. Clin. Nutr..

[53] McNamara D.J. (2002). Eggs and heart disease risk: Perpetuating the misperception. Am. J. Clin. Nutr..

[54] Howard B.V., Knowler W.C., Vasquez B., Kennedy A.L., Pettitt D.J., Bennett P.H. (1984). Plasma and lipoprotein cholesterol and triglyceride in the Pima Indian population: Comparison of diabetics and nondiabetics. Arteriosclerosis.

[55] Pelletier X., Thouvenot P., Belbraouet S., Chayvialle J.A., Hanesse B., Mayeux D., Debry G. (1996). Effect of egg consumption in healthy volunteers: Influence of yolk, white or whole-egg on gastric emptying and on glycemic and hormonal responses. Ann. Nutr. Metab..

[56] Shi Z., Yuan B., Zhang C., Zhou M., Holmboe-Ottesen G. (2011). Egg consumption and the risk of diabetes in adults, Jiangsu, China. Nutrition.

[57] Zhang J.G., Wang Z.H., Wang H.J., Du W.W., Su C., Zhang J., Jiang H.R., Zhai F.Y., Zhang B. (2015). Dietary patterns and their associations with general obesity and abdominal obesity among young Chinese women. Eur. J. Clin. Nutr..

[58] Shi Z., Yuan B., Hu G., Dai Y., Zuo H., Holmboe-Ottesen G. (2011). Dietary pattern and weight change in a 5 year follow up among Chinese adults: Results from Jiangsu nutrition and health study. Br. J. Nutr..

[59] He Y., Li Y., Lai J., Wang D., Zhang J., Fu P., Yang X., Qi L. (2013). Dietary patterns as compared with physical activity in relation to metabolic syndrome among Chinese adults. Nutr. Metab. Cardiovasc. Dis..

[60] Brown K.H., Rivera J.A., Bhutta Z., Gibson R.S., King J.C., Lonnerdal B., Ruel M.T., Sandtrom B., Wasantwisut E. (2004). International Zinc Nutrition Consultative Group (IZiNCG) technical document #1. Assessment of the risk of zinc deficiency in populations and options for its control. Food Nutr. Bull..

[61] Vashum K.P., McEvoy M., Shi Z., Milton A.H., Islam M.R., Sibbritt D., Patterson A., Byles J., Loxton D., Attia J. (2013). Is dietary zinc protective for type 2 diabetes? Results from the Australian longitudinal study on women’s health. BMC Endocr. Disord..

